# Emerging roles of tRNA modification-mediated codon-specific translational reprogramming in cancer biology

**DOI:** 10.1038/s41419-025-08234-3

**Published:** 2026-01-07

**Authors:** Hanwei Wang, Junsi Zhang, Cen Jiang, Sunwang Xu

**Affiliations:** 1 Department of Thyroid and Breast Surgery, The First Affiliated Hospital, Fujian Medical University, Fuzhou, China; 2 Central Laboratory, Fujian Medical University Union Hospital, Fuzhou, China; 3 Department of Thyroid and Breast Surgery, National Regional Medical Center, Binhai Campus of the First Affiliated Hospital, Fujian Medical University, Fuzhou, China; 4 Fujian Provincial Key Laboratory of Precision Medicine for Cancer, Fuzhou, China

**Keywords:** Cancer, Diseases

## Abstract

Cancer has become a leading cause of mortality worldwide, with alarming increases in incidence and mortality rates. Emerging evidence suggests that tRNA modification enzymes play a crucial role in cancer development by modulating codon-specific translation. In this review, we focus on 18 tRNA modification enzymes and elucidate their mechanisms of action and roles in disease. We highlight the functions and mechanisms of seven tRNA regulators that mediate favorable tRNA translation in tumorigenesis and cancer progression, providing deeper insights into their clinical potential as cancer-related biomarkers and prognostic indicators. These findings emphasize the need for further investigation into the therapeutic potential of tRNA modification enzymes in cancer management and their potential application in personalized cancer therapy and diagnostics.

## Facts

- tRNA modifications mediate codon-specific translation, thereby exerting a pivotal influence on tumor initiation and progression.
- There are cancer specific differences in codon-specific translation, which not only exist in different types of cancer, but also in different subtypes.
- The substrate specificity and mechanistic axes of the promising ALKB, TRMT, NSUN and other emerging enzyme families remain to be elucidated.
- tRNA modifying enzymes have shown great potential as biomarkers and targeted therapeutic drugs.

## Introduction

Globally, the incidence of cancer is steadily increasing, representing a growing public health burden. A report released by the International Agency for Research on Cancer (IARC) of the World Health Organization in February 2024 stated that there were 20 million new cancer cases and 9.7 million cancer deaths worldwide in 2022. It is estimated that by 2050, the number of new cancer cases globally will exceed 35 million [1].

Studies have shown that regulating the malignant biological behavior of tumors can effectively inhibit their occurrence and development. The biological behavior of tumors mainly includes self-renewal, proliferation, invasion, and metastasis, which promote the continuous proliferation and spread of tumor cells to other parts of the body. Various factors, such as the tumor microenvironment, cell signaling pathways, and epigenetics, influence malignant behavior.

With increasing research, the influence of epigenetic factors on tumors has become increasingly valued. Epigenetic factors include DNA and RNA methylation modifications, noncoding RNA regulation, chromosome remodeling, and histone modifications. Since the first record of RNA modifications in the 1950s, more than 170 types of RNA modifications have been discovered [2]. Among them, methylation accounts for approximately two-thirds of RNA modifications. RNA methylation sites include most nitrogen atoms, in addition to the oxygen atom of ribose 2’OH, the carbon atom at position 5 of pyrimidine, and the second and eighth carbon atoms of adenosine, which also undergo methylation reactions. The types of RNA methylation also differ and include m^6^A, m^1^A, m^5^C, m^1^G, m^7^G, m^5^U, etc. Research has shown that m^6^A methylation plays an important role in tumors; for example, METTL16, an enzyme responsible for RNA m^6^A modification, interacts with EIF3A/B in hepatocellular carcinoma (HCC) cells, mainly through the R1 and R2 regions of METTL16. This interaction promotes mRNA translation and the survival and proliferation of HCC cells [3].

Previous studies have mostly emphasized mRNA modification as a key regulatory factor determining tumor occurrence and development [4]. Recent emerging evidence suggests that some tRNA modifications play crucial roles in regulating tumors [5]. The tRNA modifications found thus far include the base modifications m^1^A, m^3^C, m^5^C, 2’-o-methylribose, Ψ, D, and I and 2’-O-methylated and phosphorylated ribose modifications; tRNA modification may participate in regulating the fate of cancer cells by controlling codon-specific translation [6, 7]. Subsequent research has indeed confirmed these views [8].

However, as a form of epigenetic modification, the mechanism of action of many tRNA modifications in tumors is still unclear [9]. In this review, we provide the latest information on common tRNA modifications by analyzing the structure of tRNAs, indicating that tRNA modifications need to be studied through tRNA enzyme modification-mediated codon-specific translation to influence diseases and cancers, providing more insights into their clinical potential as cancer-related biomarkers, prognostic markers, and more.

## Types of tRNA modification

### Nucleobase modification

#### N⁶-methyladenosine (m⁶A)

N⁶-methyladenosine is a modified nucleotide formed by adding a methyl group to the N⁶ position of adenosine (A). This modification is the most common internal posttranscriptional modification in animals, plants, and yeast [10–13]. It is widely present in various RNA molecules, such as mRNA, tRNA, lncRNA, and hnRNA (heterogeneous nuclear RNA). In hnRNA and mRNA, it occurs mainly on the 6th nitrogen atom of adenine and appears in the clear sequence -N1-(GA)-m6A-C-N2- [14]. There are many types of m^6^A; for example, METTL3 and METTL14 form stable coordination compounds that methylate m^6^A [15].

**Fig. 1:**
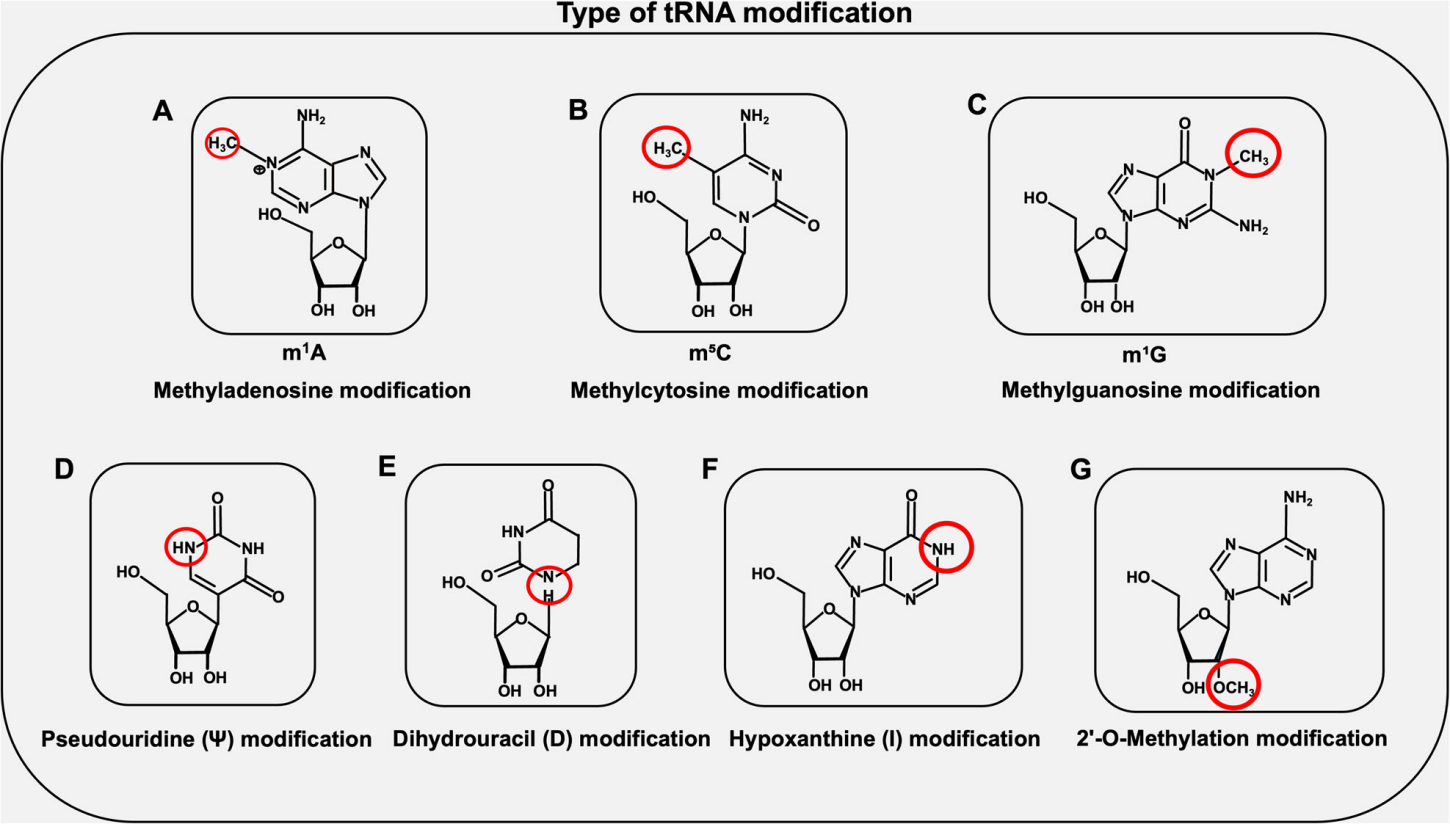
Type of tRNA modification. Several types of tRNA modifications: **A** Methyladenosine modification, Taking m^1^A modification as an example. **B** Methylcytosine modification, Taking m^5^C modification as an example. **C** Methylguanosine modification, Taking m^1^G modification as an example. **D** Pseudouridine modification. **E** Dihydrouracil modification. **F** Hypoxanthine modification. **G** 2’-O-Methylation modification.

However, m^6^A modification is very rare in tRNAs, and the common adenosine methylation modifications in tRNAs are m^1^A and m^2^A, which occur mainly at the N¹ and N² positions of adenosine. These modifications serve many functions: m^1^A can promote antitumor immunity in CD8+ T cells by enhancing the translation of ATP citrate lyase [16]. m^2^A in tRNA enhances protein translation by decoding m^2^A-tRNA-dependent codons [17]. (Fig. 1)

m^6^A RNA methylation modulates tumorigenesis, inflammation, and aging by orchestrating posttranscriptional circuits that govern oncogenic signaling, DNA repair capacity, and mitochondrial stress responses [18]. Hypomethylation drives endometrial carcinogenesis via derepression of AKT signaling [19]. METTL3-mediated hyper-methylation promotes gastric cancer through the HDGF axis, positioning METTL3 as both prognostic biomarker and therapeutic target [20]. YTHDF2-dependent m^6^A recognition destabilizes inflammation-related transcripts, thereby tuning the intensity of immune responses [21]. Overall, m^6^A epitranscriptomic rewiring couples cellular stress adaptation to clinical phenotypes across cancer, immune, and aging.

#### 5-Methylcytosine (m⁵C)

5-Methylcytosine (m^5^C) is a modified base formed by the addition of a methyl group (-CH3) to the 5th carbon of the pyrimidine ring of cytosine. It is a common RNA modification that occurs in the rRNA, tRNA, noncoding RNA and ribosomal RNA of plants and animals and can help the body cope with changes in internal and external environments [22–26]. In tRNA, m^5^C is catalyzed by the NOL1/NOP2/sun (Nsun) family and DNA methyltransferase member 2 (DNMT2, TRNA aspartate methyltransferase 1 or TRDMT1) [27–29].

m⁵C RNA methylation is involved in diverse human diseases, including cancer, viral infection, cardiovascular disease, and neurological disorders [30]. Aberrant m⁵C modification drives intrinsic resistance to gefitinib in EGFR-mutant NSCLC through the NSUN2/YBX1/QSOX1 axis, providing new insight into lung cancer resistance mechanisms and therapeutic strategies [31]. Targeting NSUN2 or m⁵C modification may suppress HBV replication and prevent related liver disease [32]. m⁵C modification also contributes to cardiovascular pathogenesis by modulating gene expression and mitochondrial dysfunction, highlighting novel molecular mechanisms and potential therapeutic targets in cardiovascular disease [33].

#### 1-Methylguanosine (m¹G)

1-Methylguanosine (m^1^G) modification involves the addition of a methyl (-CH3) group to the first nitrogen atom (N1) of guanosine (G) to ensure the correct pairing of codons and anticodons during translation. This modification is widely present in various types of RNA and has important effects on the structure, function, and physiological activities of RNA [34]. For example, in yeast cell tRNA, the formation of m^1^G modifications is closely related to the open reading frame YOL093w (named TRM10). The Trm10p protein encoded by TRM10 is responsible for the methylation of the 9th G of tRNA. Therefore, m^1^G modification may be crucial for maintaining the normal structure and function of tRNA [35]. In *E. coli* tRNA, m^1^G modification is located at position 37 of the tRNA sequence, and m ^1^G37 is necessary for the terminal methylation of U at position 34 (forming mcmo^5^U34) in tRNA (Pro) [36].

In cancer, m^1^G modification has multiple functions. The m^1^G methylation level of mitochondrial tRNA in tumor tissue is significantly altered; this abnormal modification may be closely related to the occurrence and development of tumors and is expected to serve as an important biomarker or potential therapeutic target in tumor research [37, 38]. This modification can also threaten human health, such as by inducing disease of the human reproductive system [39].

#### Pseudouridine (Ψ)

Pseudouridine (Ψ) modification is a common RNA epigenetic modification, which refers to the substitution of uracil (U) in RNA molecules with pseudouracil (Ψ). This modification is widely present in various RNA molecules, including mRNA, noncoding RNA, and rRNA, and has a significant effect on the function and stability of RNA [40].

For example, in embryonic stem cells, the Ψ modification of tRNA is mediated by the “writing enzyme” PUS7, which modifies tRNA and its derived small fragments (tRFs) to regulate protein synthesis. In addition, Ψ modification plays a crucial role in the development and functional maintenance of hematopoietic stem cells and is closely related to the occurrence and development of hematological diseases [41]. Through its abnormal modification (mediated by PUSs), PSI affects RNA function, participates in the occurrence and development of cancer, and can serve as a potential biomarker for cancer diagnosis and prognosis. Targeted PUS therapy strategies provide new research ideas for cancer treatment [42, 43].

#### Dihydrouracil (D)

Dihydrouracil (D) is present mainly in the D loop of tRNA and is catalyzed by Dus. Its modification process involves regions such as the D loop, T loop, and anticodon stem. The presence of D helps to stabilize the overall structure of tRNA, increase its flexibility, and promote accurate recognition of codons and transport of amino acids during translation, ensuring the accuracy and efficiency of translation [44]. D modification is located on the dihydrouracil arm of tRNA and may play an important role in the later stages of tRNA evolution. It helps stabilize specific structural domains of tRNA, affecting its interactions with ribosomes, aminoacyl tRNA synthetases, and mRNA and enhancing the specificity and accuracy of tRNA function, thereby ensuring efficient protein synthesis [45].

#### Hypoxanthine (I)

The modification of hypoxanthine (I) involves an enzymatic reaction that inserts hypoxanthine into the swing position of the tRNA anticodon, resulting in the formation of inosine. This modification significantly expands the codon recognition range of tRNA, enabling it to more flexibly interpret the genetic code in protein synthesis, thereby improving the efficiency and accuracy of protein synthesis and maintaining normal physiological functions [46].

### Ribose modification

#### 2’-O-methylation

2 ‘- O-methylation refers to the substitution of the hydroxyl group (-OH) at the 2’ position of RNA nucleotides with a methyl group (-CH3), thereby altering the chemical structure and functional properties of RNA. This modification is widely present in various RNA molecules, such as mRNA, tRNA, rRNA, and miRNA, and significantly affects gene expression regulation, protein synthesis, and other biological processes [47]. The 2’-O-methylation of tRNA affects mainly its structural stability and function. This modification maintains the secondary and tertiary structure of tRNA, ensuring correct folding and spatial conformation, thereby promoting interactions between tRNA and ribosomes and between aminoacyl tRNA synthetases, thereby increasing codon recognition and amino acid loading efficiency and improving the accuracy and efficiency of protein synthesis [48]. In addition, it plays many roles in immune and other diseases. In T cells, interferon-induced ISG20 impairs the reverse transcription of hypomethylated HIV-1, indicating that 2′-O-methylation directly antagonizes ISG20-mediated antiviral activity, facilitating HIV-1 evasion of host restriction and promoting viral replication [49, 50].

## Function of tRNA modification enzymes

### ALKBH8

The ALKB (ALKB homologs) family belongs to the 2-oxoglutarate (2OG)- and iron (Fe(II))-dependent dioxygenase superfamily and is widely present in mammals, plants, and viruses. The family members include ALKBH1 to ALKBH8 and FTO [51–53]. The AlkB family repairs DNA damage through oxidative demethylation and can repair alkylated damage, such as methyladenine at N1 and methylcytosine at N3, through oxidative dealkylation [54–57].

ALKBH8 is a tRNA methyltransferase with DNA repair and tRNA modification activities that also functions as an m^6^A demethylase [58]. ABH8 is the most unique among AlkB homologous genes in other mammals because of its fusion of the RNA recognition motif (RRM) and S-adenosyl-l-methionine (SAM)-dependent methyltransferase (MT) motif to the amino and carboxyl termini of the AlkB oxygenase motif, respectively [59].

The catalytic activity of ALKBH8 is mediated by two key structural domains: the C-terminal methyltransferase (MT) domain and the central AlkB oxygenase (Ox) domain. The MT domain can methylate CM5U in tRNA to mcm^5^U, while the Ox domain further modifies it to (S)-5-methoxycarbonylhydroxymethyluridine ((S)-mcm^5^U) and has stereoselectivity for the enzymatic hydroxylation of mcm^5^U [60–62]. ALKBH8 and Trm9 have similar functions and can catalyze the conversion of cm^5^U to mcm^5^U. tRNAArg (UCU) and tRNAGlu (UUC) have been shown to be two uridines containing swing tRNAs associated with the ALKBH8 complex. Therefore, ALKBH8 regulates protein translation by modifying the swing uridine of tRNAs [59, 63].

The mechanism of action of ALKBH8 is complex, and its target genes are not fully understood; however, its biochemical characteristics indicate that it is associated with various diseases. For example, the loss of ALKBH8 function may interfere with the tRNA modification process, thereby affecting brain function and leading to intellectual developmental disorders [61, 64, 65]. In addition, ALKBH8 plays important roles in protecting against acetaminophen (APAP) toxicity, regulating selenoprotein levels, aging, stress response gene regulation, mitochondrial weight programming, and maintaining neurological function [66–68]. ALKBH8 also promotes the growth of bladder cancer by regulating survivin expression, but its relationship with tRNA levels has not been confirmed [69].

### DNMT2

DNMT2 (DNA methyltransferase 2) belongs to the DNA methyltransferase family and has an evolutionary history older than that of DNMT1 and DNMT3. It is widely present in various organisms, including plants, fungi, insects, fish, amphibians, birds, and mammals [70]. DNMT2 has multiple domains, including a target recognition domain (TRD) and a target recognition extension domain (TRED). Its domain is similar to that of prokaryotic m^5^C MTase and is involved in DNA damage recognition, recombination, and mutation repair [71–73].

DNMT2 plays an important role in tRNA modification, is capable of methylating tRNA (Asp GTC), tRNA (Val AAC), and tRNA (Gly GCC), and plays a role in the biosynthesis of tRNA-derived small RNAs [74–76]. During the formation of tRNA m^5^C38, quinine (Q) can increase DNMT2 activity [77–79]. DNMT2 recognizes the C32U33 (G/I) 34N35 (C/U) 36A37C38 motif in the tRNA anticodon loop, U11 in the D stem A24, and the correct size variable loop, selectively modifying tRNA [80, 81].

DNMT2 also participates in the regulation of protein translation. For example, DNMT2-mediated tRNA Asp methylation achieves posttranscriptional regulation by controlling the synthesis of target proteins containing polyaspartic acid sequences [82]. The DNMT2-mediated loss of tRNA Asp (GTC) C38 methylation leads to the downregulation of proteins with an Asp GAC codon bias [83]. In addition, DNMT2 and NSUN2 jointly promote the stability of tRNA and protein synthesis [84]. At the cellular level, DNMT2 silencing can induce oxidative stress and DNA damage in human fibroblasts and regulate cell proliferation and lifespan, and its regulation may become a potential anticancer strategy [85, 86]. In addition, DNMT2 also participates in antiviral defense mechanisms [83].

### Mettl family

#### METTL1

METTL1 is a methyltransferase with an S-adenosylmethionine (SAM)-binding domain. Its characteristic 7-β chain catalytic domain enables it to modify DNA, RNA, and proteins [87, 88].

METTL1 works synergistically with WDR4 to introduce m^7^G modification at position 46 of tRNA, which is crucial for the normal function of tRNA and is the most common methylation site in prokaryotic and eukaryotic organisms [89, 90]. m^7^G modification not only affects overall methylation levels but also dynamically regulates internal methylation sites, especially in the GA/GG enrichment region of the 5’-untranslated region (UTR), as well as significantly increasing and increasing the accumulation of m7G modifications in coding sequences (CDSs) and 3’-UTRs under oxidative and heat stress [91–94].

WDR4 regulates the activity of METTL1 through two mechanisms, the first of which is by enhancing the binding affinity between METTL1 and SAM. The N-terminus of METTL1 regulates the methylation activity of m^7^G46 by coordinating SAM binding, RNA binding, and conformational changes in tRNA and the METTL1-WDR4 complex [95]. The second is by providing a scaffold for RNA binding, promoting the correct orientation of substrate tRNA and thereby achieving efficient catalysis [96]. In addition, METTL1 can be phosphorylated and inactivated by PKB and RSK both in vitro and in cells [97].

**Fig. 2:**
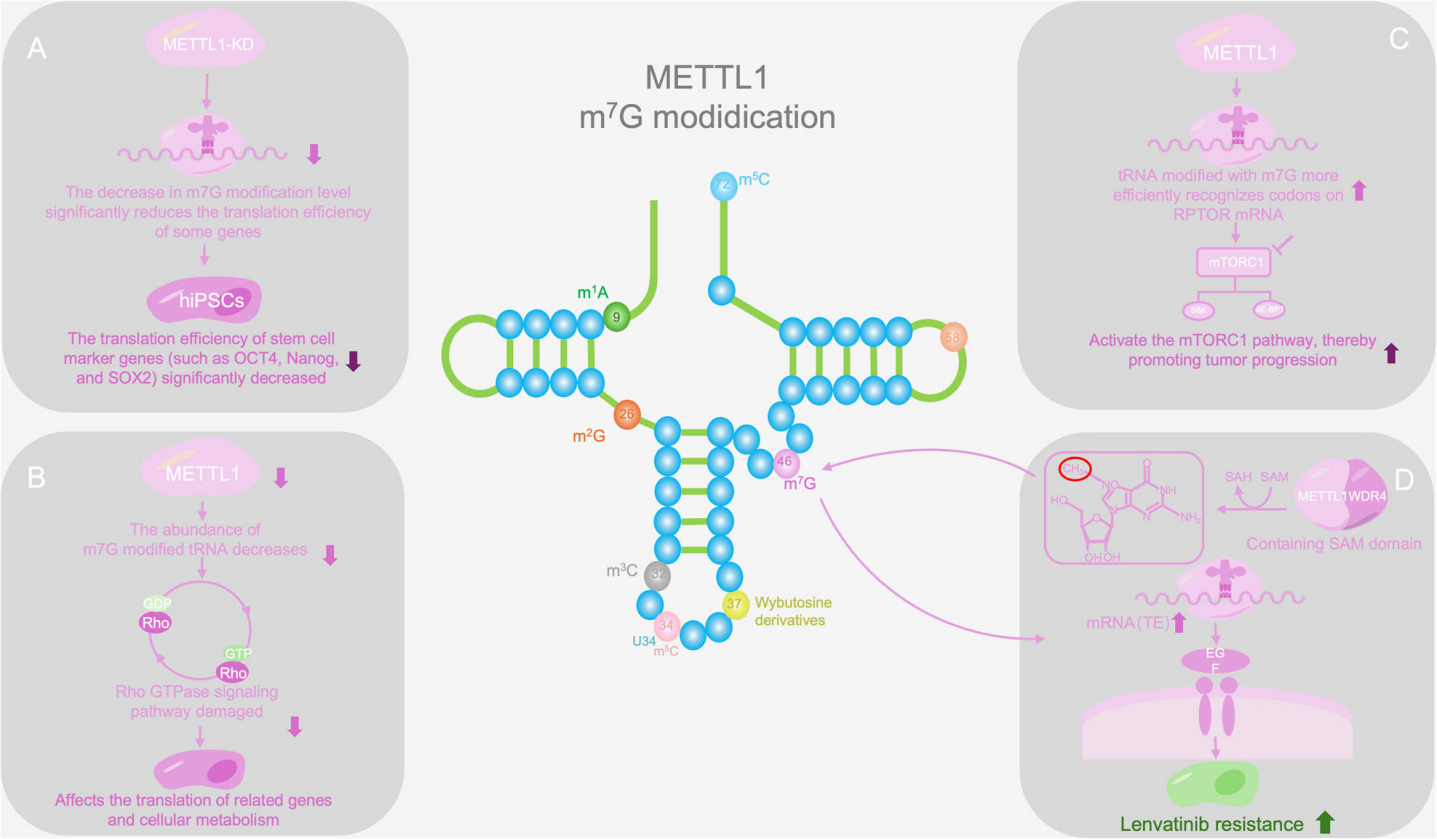
m^7^G modification of METTL1. **A** METTL1 silencing can reduce the translation efficiency of hiPSC marker genes in stem cells. **B** Knockout of METTL1 leads to a decrease in m^7^G modification, resulting in damage to the Rho GTPase signaling pathway. **C** The m^7^G modification of METTL1 can activate the mTORC1 pathway and promote tumor development. **D** METTL1 affects the EGFR signaling pathway through m^7^G modification, regulates codon translation efficiency, and influences lenvatinib resistance.

METTL1 plays important roles in various biological processes. METTL1-mediated m^7^G modification can maintain normal mRNA translation, self-renewal and differentiation in embryonic stem cells and, furthermore, can affect fertility by regulating tRNA homeostasis levels and the translation efficiency of genes required for spermatogenesis [98, 99]. In addition, METTL1-WDR4-catalyzed tRNA m^7^G46 modification can regulate translation, thereby affecting aging [100]. The conditional deletion of METTL1 or missense mutation of WDR4 can impair endochondral bone formation and accumulation, the mechanism of which is related to METTL1 knockout, which reduces the abundance of m^7^G-modified tRNA and inhibits the translation of mRNA related to the cytoskeleton and Rho GTPase signaling [101, 102]. METTL1 silencing can reduce the translation efficiency of hiPSC marker genes in stem cells and provides new ideas for the treatment of neurological and vascular diseases via the inhibition of mesodermal differentiation and angiogenesis [103]. METTL1 is also a potential therapeutic target, and its silencing can inhibit the growth of tumor cells [104] (Fig. 2).

METTL1 enhances the translation of GADD45A and RB1 through an m⁷G dependent, codon-specific mechanism that increases tRNA-m⁷G levels, thereby inducing G₂/M arrest and suppressing breast cancer cell proliferation while increasing the antitumor efficacy of CDK4/6 inhibitors such as abemaciclib. Given that CDK4/6 inhibitors are primarily employed in the luminal A/B (ER⁺) breast cancer subtype, METTL1 appears to be closely associated with this subtype. In contrast, the mRNA expression levels of METTL1 and WDR4 are lower in the HER2⁺ and triple-negative subtypes than in luminal A/B tumors and normal breast tissue. Collectively, these findings indicate that METTL1 may exert distinct effects across different breast cancer subtypes [105, 106]. Whether tRNA modifications at specific codons are correlated with tumor subtypes and the precise molecular mechanisms through which they participate in tumorigenesis and progression of these subtypes warrant further investigation.

#### METTL6

METTL6 is an S-adenosylmethionine-dependent methyltransferase that can specifically modify tRNA (Ser) to resist N(3)-methylcytosine (m^3^C) at the 32nd nucleotide of the copper ring [107]. METTL6 shares homology with Trm141 in *Streptococcus pyogenes* and targets serine tRNA [108]. METTL6 can modify tRNA through its unique structural domain, m^3^C-RBD (m^3^C-specific RNA binding domain). This domain consists of an N-terminal region, an internal insertion, and an extended hairpin with a central β fold, forming a positively charged groove that accommodates the anticodon stem through nonspecific electrostatic contact with the base while recognizing the modified C32 through base-specific contact [109].

**Fig. 3:**
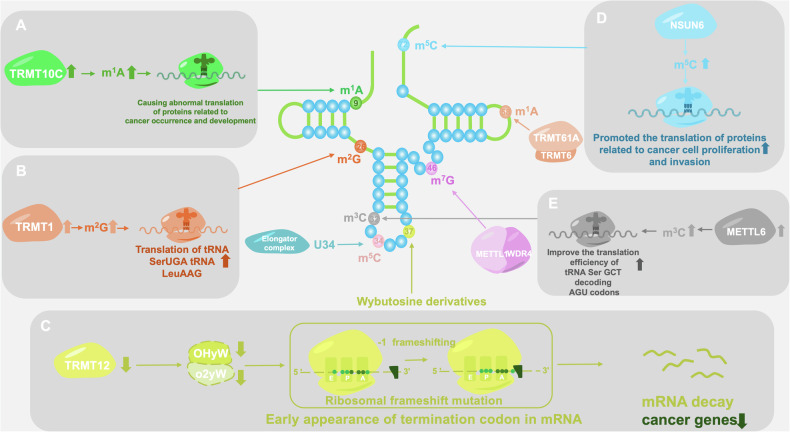
Various enzyme mediated tRNA modifications. tRNA modifying enzymes affect codon-specific translation: **A** TRMT10C promotes cancer progression via m1A modification. **B** Trm1 modifies tRNA SerUGA and tRNA LeuAAG. **C** TRMT12 inactivation reduces tRNA modifications, causing ribosomal -1 frameshifts. **D** NSUN6 upregulation increases tRNA m^5^C modification, enhancing translation of cancer-related proteins. **E** METTL6-mediated m^3^C modification boosts translation efficiency of tRNA SerGCT decoding AGU codons.

The modification function of METTL6 involves codon bias, which can improve the translation efficiency of tRNA Ser GCT decoding AGU codons, thereby regulating the cell cycle and DNA damage response [110]. In addition, METTL6 plays important roles in various cellular functions. For example, METTL6 knockout significantly reduces the sensitivity of lung cancer cells to cisplatin, indicating its potential role in chemotherapy resistance [111]. In hepatocellular carcinoma (HCC), METTL6 depletion inhibits cell growth, colony formation, migration, invasion, and cell adhesion and reduces the expression levels of cell adhesion proteins such as ITGA1, SPON1, and CLDN14 [112]. The expression of METTL6, a tumor-associated gene (TRG), is strongly correlated with the prognosis of patients with HCC and can serve as a potential prognostic indicator, providing new directions for the treatment strategies of patients with HCC [113] (Fig. 3).

### PUS1

Pseudouridylate synthase (PUS) catalyzes pseudouridine (Ψ) modification, which is an RNA modification that plays an important role in cellular function [114]. Pseudouridylate synthase 1 (PUS1) is a key member of this family, and its functions vary significantly among different organisms. Yeast Pus1 is a multisite-specific enzyme that synthesizes Ψ 34 and Ψ 36 in tRNAIle (UAU). In contrast, Pus1 (cmPus1) in *Cyanidioschyzon merolae* only catalyzes the Ψ modification at positions 34, 36, and/or 55 in certain specific introns containing precursor tRNA (UAU) variants [115].

PUS1 plays important roles in various biological processes. Pseudouridine modification is critical for the regulation of tRNA homeostasis, cytoplasmic translation, and erythropoiesis [116]. PUS1 gene mutation can lead to the loss of pseudouridine modification in mitochondrial tRNA, resulting in mitochondrial dysfunction and impaired protein synthesis [117]. In addition, PUS1 may participate in DNA repair, E2F targeting, MYC targeting, and the G2M checkpoint and promote malignant transformation in non-small cell lung cancer (NSCLC) through mcm^5^ or XPO1 [118]. PUS1 silencing can significantly inhibit the proliferation and invasion of breast tumors and may serve as a potential diagnostic biomarker for various cancers, including renal cell carcinoma (RCC) [119, 120]. PUS1 may also be involved in metabolic pathways, mitochondrial function, nonalcoholic fatty liver disease (NAFLD), and important oncogenic pathways and can be used as a clinical diagnostic biomarker for sepsis [121, 122]. In addition, the homozygous C656T mutation in the PUS1 gene is one of the pathogenic mutations in MLASA syndrome [123].

### TRMT family

#### TRM1

TRM1 (tRNA methyltransferase 1) is a zinc ion binding, S-adenosylmethionine-dependent methyltransferase involved in tRNA processing, with RNA binding and tRNA (guanine N2) methyltransferase activity, and is mainly responsible for modifying m^2^G in tRNA. TRM1 exhibits substrate specificity and multisite modification ability in different organisms. For example, TRM1 in A. aeolicus not only catalyzes the methylation of G26 to generate m^2^G but also catalyzes the methylation of G27 to generate m^22^G [124]. In addition, endogenous and overexpressed Trm1 significantly modifies tRNA SerUGA and tRNA LeuAAG, indicating a preference for specific tRNA substrates. Mechanistically, Trm1 recognizes the secondary and tertiary structures of tRNA through domain specificity and targets the precursor tRNA that has been terminally processed and contains introns. This combination does not rely on catalytic activity. The binding of precursor tRNA to La inhibits the methylation of Trm1, while the substrate binds weakly to the RNA chaperone protein La because of its 3’ end sequence characteristics, resulting in reduced inhibition. At the same time, the RNA annealing partner activity of Trm1 promotes the formation of a preferred substrate conformation suitable for G26 modification, thereby efficiently completing the modification [125]. Among Archaeans, the TRM1 enzyme of *Pyrococcus abyssi* is a homologous tetramer with site specificity that can catalyze m^1^A modifications at positions 57 and 58 of certain tRNA T loops, whereas the TRM1 enzyme of *Pyrococcus furiosus* can be expressed and function in *Escherichia coli* [126–128].

TRM1 plays important roles in various biological processes. It may participate in the regulation of neuronal function after birth and maintain cell proliferation and cell survival under oxidative stress [129, 130]. These functions indicate that TRM1 plays important roles in cellular physiology and stress responses.

TRM1 generally exists in the human body in the form of its homolog, which is TRMT1. Its function and function are similar to those of TRM1. TRMT1 can methylate all tRNAs known to contain guanosine at position 26, catalyzing the formation of N2,N2-dimethylguanosine (m^2,2^G). Mutations in TRMT1 may lead to tRNA modification defects, which in turn can cause related diseases [131].

#### TRM4/NSUN2

TRM4 is a tRNA methyltransferase that uses S-adenosylmethionine (AdoMet) as a methyl donor to catalyze cytosine methylation on tRNA molecules to generate m^5^C modifications [132, 133]. m^5^C modification is widely present in RNA and affects mainly biological processes, such as RNA stability, splicing, and nuclear cytoplasmic transport. TRM4 acts as a tRNA m^5^C methyltransferase in brewing yeast and *Methanococcus jannaschii* specifically by modifying the C34 site in yeast pre-tRNA Leu (CAA) [132–134].

**Fig. 4:**
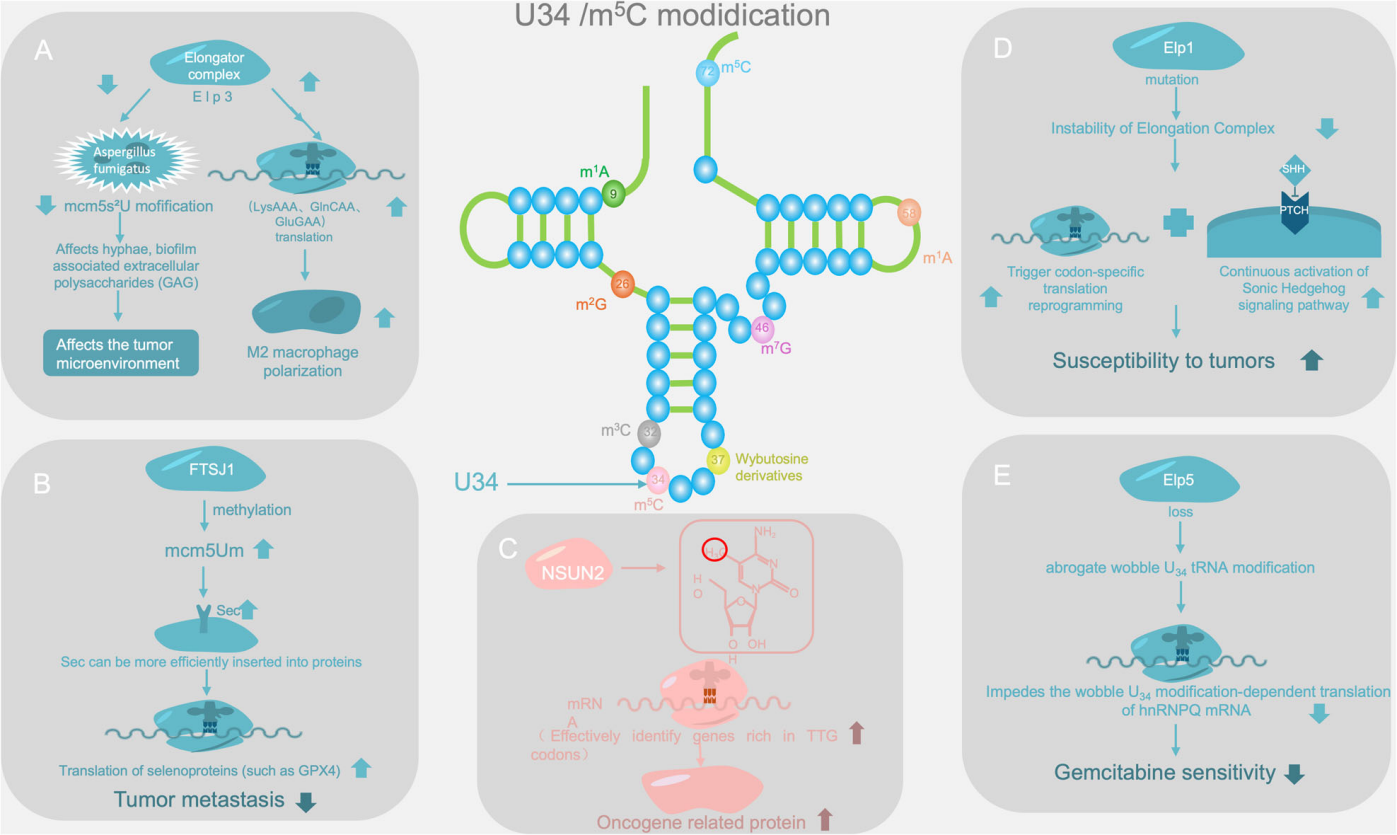
U34/m^5^C modification. **A** ELP3 affects cancer development by influencing the tumor microenvironment and specific codon translations. **B** FTSJ1 mediated methylation modification enhances translation of related selenoproteins and inhibits cancer cell metastasis. **C** NSUN2 regulates m⁵C modification, selectively translating mRNAs with TTG codons and promoting cancer-related protein synthesis. **D** Elp1 mutation leads to codon specific translation, activates the PTCH signaling pathway, and affects tumor susceptibility. **E** Elp5 deficiency affects U_34_ modification and reduces sensitivity to gemcitabine.

TRM4 plays important roles in various biological processes. In yeast cells, TRM4 participates in codon bias-based gene expression regulation by modulating tRNA modifications. Under oxidative stress conditions in yeast cells, TRM4 regulates tRNA modification, selectively translates mRNAs containing more TTG genes, and can also alter ribosome composition [135]. In addition, TRM4 is associated with sensitivity to 5-fluorouracil and has genetic interactions with other tRNA-modified genes, such as PUS1, PUS4, PUS7, and TRM2 [136]. The absence of TRM4 triggers the RTD (ribosome rescue) pathway, affecting the normal lifecycle of tRNA. TRM4 also synergizes with TRM8 to regulate cell growth and predict the accumulation of protein–RNA complexes after homocysteine mutations [137–139] (Fig. 4).

NSUN2 is a mammalian homolog of yeast TRM4, belongs to the RNA methyltransferase family and is encoded by the human NSUN2 gene. It adds a methyl group to the C5 position of the RNA cytosine base through the catalytic mechanism of homocysteine, resulting in m^5^C modification. This modification is widely present in tRNA, rRNA, mRNA, and various types of noncoding RNA and is highly important for the structural stability, metabolic regulation, and functional performance of RNA [84, 140, 141].

NSUN2 plays crucial roles in various biological processes. Research has shown that NSUN2 deficiency can lead to reduced efficiency of glycine codon-specific translation, which in turn affects protein synthesis, synaptic transmission in mature neurons, and other complex behaviors. In addition, NSUN2 may affect intellectual development and disease occurrence through genetic mutation or abnormal methylation [141–144]. In cancer, abnormal NSUN2 expression is associated with the occurrence and development of various cancers, such as oral cancer and rectal cancer [145].

#### TRM9

TRM9 is a product of the yeast YML014w gene, which can catalyze the esterification reaction of uridine nucleotides and form specific modifications in tRNA [146]. TRM9 is mainly responsible for catalyzing the modification of mcm^5^ and mcm^5^s^2^ in tRNAArg (UCU) and tRNAGlu (UUC), which can regulate the translation selectivity of proteins. For example, Trm9 deficiency leads to the loss of the mcm^5^ and mcm^5^s^2^ modification of tRNAArg (UCU) and tRNAGlu (UUC), resulting in a decrease in their pairing efficiency with AGA and GAA codons. It is difficult for unmodified tRNA to quickly match the AGA and GAA codons on mRNA during translation, causing ribosome arrest, translation deceleration, and reduced protein synthesis [147]. In addition, TRM9-mediated specific tRNA modification can enhance codon-specific translation extension and increase the levels of key damage response proteins such as Yef3, Rnr1, and Rnr3 [146].

In terms of tumor biology, the expression of the human homolog hTRM9L of TRM9 is significantly downregulated in a variety of cancers, including testicular cancer, cervical cancer and bladder cancer. The absence of hTRM9L may increase the sensitivity of tumor cells to drugs that induce translation errors, making hTRM9L a potential target for treating hTRM9L-deficient tumors. hTRM9L also exerts antitumor effects by inhibiting tumor growth, regulating the cell cycle, and affecting the hypoxic response [148]. However, research on the specific mechanism through which TRM9 affects tumors is still insufficient and is worth exploring.

#### TRMT2A

Human tRNA methyltransferase 2 homolog A (hTRMT2A) is an enzyme that can bind and methylate RNA with low specificity. hTRMT2A contains multiple domains, including an RNA-binding domain (RBD), a central domain, and a fusion domain, which play synergistic roles in binding to tRNA and jointly participate in the interaction between hTRMT2A and tRNA. The uridine (U) at position 54 of tRNA is crucial for the binding and methylation activity of hTRMT2A, and its specific recognition mechanism enables hTRMT2A to accurately recognize and modify its target tRNA molecule. hTRMT2A plays an important role in maintaining translational fidelity, and loss or impairment of its function may lead to an increase in protein synthesis errors, thereby affecting the normal physiological functions of cells [149].

In addition, hTRMT2A has multiple functions in cell biology. It can inhibit cell proliferation and cell cycle progression and is considered a potential drug target for treating polyQ disease (a neurodegenerative disease) [150, 151].

#### TRMT10A

The TRM10 family of methyltransferases is responsible for the N1 methylation of the 9th purine in the tRNA of Archaeans and eukaryotes. The human genome encodes three types of TRM10 enzymes, namely, TRM10A, TRM10B, and TRM10C [152]. Among them, TRMT10A can catalyze the methylation of adenine (A) or guanine (G) at the 9th position of tRNA, resulting in m^1^G modification, and enhance the m^6^A demethylase activity of FTO through interactions with the mRNA demethylase FTO, stabilizing unmodified mRNA [153, 154].

TRMT10A has specific substrates in different species. In humans, its substrates include tRNAGln (UUG/CUG) and tRNAiniMeth (CAU), whereas in yeast, its substrates are tRNAGly (GCC) and tRNAVal (UAC). tRNAGln is responsible for decoding CAA and CAG codons (corresponding to glutamine) in mRNA. The deletion of TRMT10A leads to functional defects in tRNAGln, which may reduce its recognition efficiency of CAA/CAG codons and affect the translation of genes containing these codons [155]. TRMT10A deficiency can lead to translational distortion, decreased postsynaptic density, and impaired synaptic plasticity and can affect learning and memory function in mice, indicating its crucial role in brain function [156].

In addition, TRMT10A gene mutation is associated with diabetes and primary microcephaly in young people. Its defects can lead to a decrease in tRNA methylation levels, induce beta-cell apoptosis, and thus affect insulin secretion [155, 157–160].

#### TRMT10C

TRMT10C is a tRNA methyltransferase and a subunit of mitochondrial ribonuclease P (RNase P). When it binds to MRPP2, it forms a stable m^1^R9 tRNA methyltransferase complex, and when it binds to MRPP3, it forms an RNase P complex responsible for the 5’ end cleavage of mitochondrial tRNA [161, 162]. TRMT10C is expressed in mitochondria and participates in the positive regulation of RNA metabolism and mitochondrial translation, which is of significance for the maturation and functional expression of tRNA [163–165].

In addition, TRMT10C is involved in various biological functions. It indirectly inhibits lung cancer growth by mediating m^7^G modification of circFAM126A [166]. TRMT10C also participates in N1 methyladenosine (m^1^A) modification, which plays a key role in various cancers, such as glioma, hepatocellular carcinoma, and renal clear cell carcinoma [167–171]. However, the specific mechanism is still unclear and worthy of further study.

#### TRMT12

TRMT12 (tRNA methyltransferase 12) is an enzyme involved in tRNA modification, and its gene is also known as TYW2 or TRM12 in yeast. TRMT12 is essential for the synthesis of wybutosine (yW) in yeast, which is an important tRNA modification that regulates mRNA translation efficiency and maintains the correct reading framework.

TRMT12 exhibits significant changes in expression in various cancers. For example, in head and neck squamous cell carcinoma (HNSCC) tissue, the expression of TRMT12 is significantly upregulated, which may be related to genetic and epigenetic changes [172, 173]. In addition, through whole-exome sequencing of tumor and nonmalignant samples from 12 patients with special peripheral T-cell lymphoma (PTCL), researchers identified TRMT12 as one of 70 genes with somatic mutations [174]. However, the specific mechanism of action of TRMT12 in cancer is still unclear and deserves further investigation, which may provide a new perspective for the study of tumor development.

#### Trmt61A

**Fig. 5:**
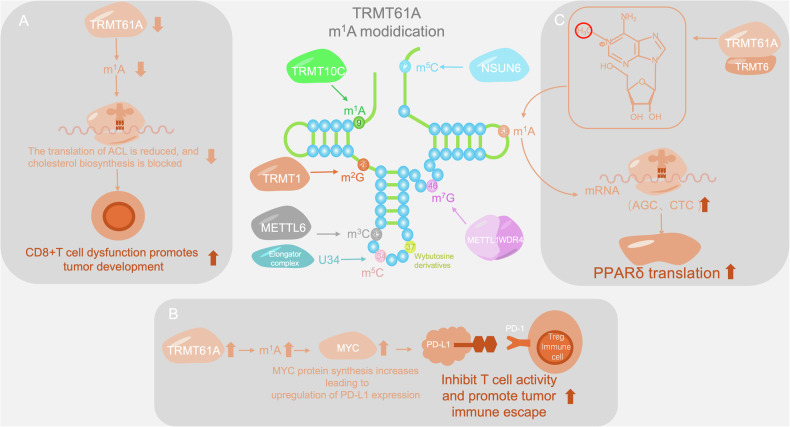
m^1^A modification of TRMT61A. Three examples of m^1^A modification of TRMT61A: **A** TRMT61A knockout reduces tRNA m¹A modification, inhibits ACL translation, and impairs CD8⁺ T cell function, promoting tumor immune evasion. **B** TRMT61A overexpression enhances tRNA m¹A modification, boosts MYC synthesis, and upregulates PD-L1, aiding tumor immune escape. **C** The TRMT61A-TRMT6 complex increases m¹A methylation, particularly on tRNAAla(AGC) and tRNAGlu(CTC), thereby boosting translation efficiency and PPARδ synthesis.

TRMT61A belongs to the tRNA methyltransferase family and methylates adenine (A) to generate N1 methyladenosine (m^1^A) [175]. This modification occurs in the nucleus, where TRMT61A and TRMT6 work together to modify specific tRNAs with m^1^A58. The m^1^A58 modification may enhance the interaction between tRNA and ribosomes and between aminoacyl tRNA synthetases, accelerate the translation process, and rapidly synthesize MYC and other key functional proteins by changing the local conformation of tRNA. MYC, as a core transcription factor that drives cell proliferation, contains high-frequency codons in its mRNA that match the modified tRNA. The m^1^A58 modification enables tRNAs to quickly decode these codons and ensure the rapid accumulation of MYC protein after T-cell activation, thereby promoting the rapid expansion of activated T cells and guaranteeing the timeliness of the immune response [176]. (Fig. 5)

In addition, TRMT6-TRMT61A complex-mediated tRNA-m^1^A58 modification plays an important role in the homeostasis of hematopoietic stem cells (HSCs) and performs nonclassical functions during HSC aging. These findings indicate that TRMT61A plays multifaceted regulatory roles in cell proliferation and stem cell aging [177–179].

#### TRMT61B

TRMT61B is a tRNA methyltransferase. In human mitochondria, it is responsible for 1-methyladenosine (m^1^A) modification at position 58 of mitochondrial tRNA (Leu (UUR)), tRNA (Lys), and tRNA (Ser (UCN)). This modification is crucial for mitochondrial protein synthesis and cellular function and plays a key role in various cancers [168].

TRMT61B regulates mitochondrial function through methylation, which may have significant implications for the pathogenesis of Alzheimer’s disease [180]. In addition, the role of TRMT61B in hepatoblastoma and melanoma has been preliminarily revealed: in the SK-MEL-103 melanoma cell line, TRMT61B knockout significantly inhibited tumor growth and metastasis and prolonged the survival of mice. Similarly, in zebrafish embryos and nude mouse models, TRMT61B inhibition slowed tumor cell proliferation. These findings suggest that targeting TRMT61B may provide new strategies for treating certain tumors [181, 182]. In addition, TRMT61B may affect protein translation through tRNA modification, thereby regulating cellular biological behavior and influencing the occurrence and development of cancer. For example, the TRMT61B gene may be related to susceptibility to breast cancer, but its specific mechanism of action in breast cancer still needs to be clarified through further research [177, 183, 184].

### TRMU

TRMU is a highly conserved mitochondrial tRNA modification enzyme that is primarily responsible for the 2-thiouridine (s^2^U) modification of three types of mitochondrial tRNA (mt tRNA) at the U34 position. This modification is crucial for maintaining the structural stability and normal function of tRNA. TRMU plays a crucial role in vertebrates, participating in the processing and modification of tRNA [185].

It has several functions; for example, TRMU variants can cause liver failure in infants [186, 187]. The functional abnormalities of TRMU are associated with various diseases. For example, mutations in the TRMU gene may lead to structural and functional abnormalities in the enzyme, especially homozygous mutations in its N-terminal conserved region (such as A10S), which can severely affect mitochondrial tRNA metabolism and ultimately result in hearing loss [188, 189]In a zebrafish model, deletion of the MTU1 gene resulted in complete disappearance of the 2-thiouridine modification of mitochondrial tRNA (Lys), tRNA (Glu), and tRNA (Gln) at position U34, indicating that TRMU plays an important role in maintaining mitochondrial tRNA modification and hearing development [190].

### TYW2

TYW2 is a tRNA modification enzyme that is involved in the synthesis of wybutosine (yW) modification in tRNA. This modification is crucial for maintaining the normal biological function of tRNA [191, 192].

In Archaeans, TYW2 plays a key role in the biosynthesis of guanosine derivatives, and its function is similar to that of homologous enzymes in eukaryotes, as it is involved in the construction of the basic skeleton of guanosine derivatives. TYW2 works synergistically with other related enzymes, such as TRM5, TYW1, TYW3, and TYW4, to determine the type and content of guanosine derivatives in Archaeans [193].

### NSUN6

The NSUN family belongs to the methyltransferase family and is the main RNA m^5^C-modifying enzyme family in eukaryotes, consisting of seven members, from NSUN1 to NSUN7. Among them, NSUN6 is mainly responsible for catalyzing the m^5^C modification of tRNA, specifically by acting on the 72nd cytosine of the tRNA amino acid arm [194]. NSUN6 recognizes the CCA end and D-stem region of tRNA through its PUA domain and precisely identifies the target base using its MTase domain, inducing conformational changes in tRNA and exposing and modifying the target base [195].

NSUN6 plays important roles in various biological processes. When NSUN6 expression is upregulated, the m^5^C modification level of tRNA increases, promoting the translation of proteins related to cancer cell proliferation and invasion. Its functional impairment can lead to abnormal m^5^C modification of tRNA and mRNA, which in turn affects the accuracy and efficiency of protein synthesis, interferes with neurological development, and leads to cognitive dysfunction [196, 197]. In addition, NSUN6 affects the synthesis of cell proliferation-related proteins by regulating tRNA methylation, thereby regulating the proliferation of pancreatic cancer cells [198]. In glioblastoma, NSUN6-mediated m^5^C modification regulates transcriptional pauses and controls the tumor response to alkylating agents through the accumulation of NELFB and transcription factor complexes (POLR2A, TBP, TFIIA, and TFIIE) at the TATA binding site [199]. NSUN6 is also involved in the development of lung cancer and other cancers and can serve as a potential therapeutic target. Its role in cognitive impairment also deserves further research [200, 201].

### Elongator complex

The elongator complex is a highly conserved multisubunit protein complex in eukaryotes that consists of six subunits, ELP1–ELP6. Among them, ELP1-ELP3 constitute the core subunit, and ELP4-ELP6 interact with the core subunit to maintain the stability and function of the complex. This complex plays important roles in gene transcription extension, tRNA modification, and other cellular processes.

Its catalytic subunit Elp3 contains an S-adenosylmethionine binding (rSAM) domain and a lysine acetyltransferase (KAT) domain, which are crucial for modification of the tRNA swing position (U34) and can promote the formation of modified nucleoside side chains such as 5-aminoformylmethyluridine (ncm⁵U₃₄), 5-methoxycarbonylmethyluridine (mcm⁵U₃₄), and 5-methoxycarbonylmethyl-2-thiouridine (mcm⁵s²U₃₄). These modified nucleosides are of significance for efficient decoding in the translation process, helping to maintain the accuracy and efficiency of protein synthesis and affecting both telomere gene silencing and the DNA damage response [202].

The elongator complex serves multiple functions. In vivo, Elp3 can promote polarization of M2 macrophages. During this process, Elp3 expression is upregulated by IL-4 and IL-13 induction. By modifying tRNA, it promotes the translation of mRNAs containing specific codons, such as LysAAA, GlnCAA, and GluGAA. These mRNA-encoded proteins participate in M2 macrophage polarization [203]. In addition, Elp3 plays an important role in the differentiation of intestinal cluster cells by regulating tRNA modification [204]. Notably, dysregulation of the tRNA modification activity of Elp3 is associated with various human diseases, including various cancers and neurodegenerative diseases, and Elp2 also participates in the regulation of these diseases [205, 206].

## Role of codon-specific translational reprogramming mediated by tRNA modification in cancer

### ALKBH8 mediates tRNA codon-specific translation in cancer

**Fig. 6:**
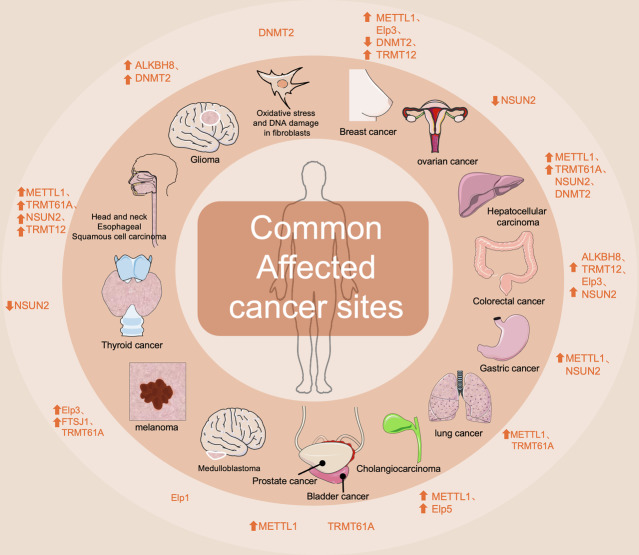
Common affected cancer sites. Modifying enzymes influence cancer occurrence and progression by affecting tRNA codon-specific translation. Multiple enzymes can impact the same cancer type. Using Springer Nature Author Services, compare the increase or decrease in the expression levels of different modifying enzymes relative to normal tissues in different tumor types.

ALKBH8 is a methyltransferase that can affect codon-specific translation by modulating tRNA modifications, thereby influencing the occurrence and development of cancer. Under oxidative stress conditions, ALKBH8 can increase the 5-methoxycarbonylmethyl-2’-methyluridine (mc^5^Um) modification of tRNA, thereby driving the expression of selenocysteine-containing ROS-detoxifying enzymes such as Gpx1, Gpx3, Gpx6, and TrxR1. This modification is crucial for maintaining the redox balance of cells. In ALKBH8-deficient (Alkbh8-/-) cells, tRNA modification is impaired, leading to translational reprogramming and codon reprogramming abnormalities, which in turn affect cell function [207]. In addition, ALKBH8 is abnormally expressed in various cancers, and its function is closely related to tumor progression. For example, in colorectal cancer (CRC), by regulating the translation of selenoproteins and affecting the oxidative stress response, ALKBH8 may become a new target for CRC treatment. ALKBH8 expression is also significantly altered in other cancers, such as glioma, suggesting its potential role in tumorigenesis and drug resistance [208–212]. (Fig. 6) (Table 1)

**Table. 1:** The main role of tRNA modifying enzymes in human cancer.

ENZYME NAME	ENZYME TYPE (MODIFYING ENZYME)	Modification Method and Target	CLINICAL RELEVANCE	THE ROLE IN TUMORS
ALKBH8	methyltransferase	Oxidative stress induction,tRNAU34	Affects the sensitivity of oxidative stress-related treatments.Middle to late stage may be a new target for CRC treatment, potentially associated with drug resistance.	Cancer: Colorectal cancer, glioma, etc.Function: Anti ROS Missing: Translation disorder Prospect:sensitization/reversal of drug resistance.
DNMT2	methyltransferase	m^5^C and C > G translocation at cytosine residues,tRNA C38	Inhibited the apoptotic signaling pathway of tumor cells, resulting in reduced sensitivity to bortezomib. The middle and late stages of cancer may become a new target for treatment.	Cancer: breast, cervical, osteosarcoma, glioblastoma, liver, fibroblast Function: Anti-ROS / modulates oxidative stress & DNA damage;Apoptosis signal blocked - bortezomib resistance Prospect: Sensitization / reversal of drug resistance.
METTL1	methyltransferase	m^7^G, tRNA G46	Related to multiple drug resistance: 1 Maintaining resistance to lenvatinib in hepatocellular carcinoma. 2. Control resistance to doxorubicin. 3. May affect OXPHOS resistance in oral squamous cell carcinoma. Early prediction of drug resistance and targeted intervention can be carried out.	Cancer: Lung, gastric, HCC, esophageal, cholangio, prostate, breast, etc. Function: Multi-drug resistance (lenvatinib, doxorubicin, OXPHOS); Early resistance prediction & targeted intervention markers Prospect: Sensitization / reversal of drug resistance.
TRMT12	methyltransferase	tRNA G37	Related to paclitaxel resistance: 1. Downregulation of TRMT12 leads to an increase in imG-14 levels, enhancing paclitaxel resistance. 2.Knocking out TRMT12 reduces the efficacy of paclitaxel. In early cancer, it may be a prognostic predictor. Mid to late stage may further enhance chemotherapy sensitivity.	Cancer: breast, head & neck squamous, rectal Function: paclitaxel resistance via imG-14 modulation; promoter hypermethylation & ribosome reading-frame error Prospect: early prognostic marker; mid-late stage chemotherapy sensitization.
TRMT61A	methyltransferase	m¹A,tRNA A58	May affect drug sensitivity related to codon decoding. Targeted therapeutic drugs can be developed during the tumor period.	Cancer: melanoma, lung, HCC, head & neck squamous, bladder, etc. Function: modulates codon-decoding–mediated drug sensitivity Prospect: targeted therapeutic drug development.
NSUN2	methyltransferase	m^5^C,tRNA C34	Affect drug sensitivity related to codon decoding. May become a diagnostic biomarker in the early stages of cancer, and develop targeted therapeutic drugs in the middle and later stages.	Cancer: thyroid, esophageal, liver, gastric, head & neck squamous, ovarian, etc. Function: secondary-structure–mediated codon decoding-oncogene translation;early diagnostic biomarker Prospect: mid-late stage targeted therapy.
elongator complex	enzyme complex	tRNA U34	Affects the tumor microenvironment, interacts with the human immune system, and is associated with multiple drug resistance:: 1. In gallbladder cancer, patients with low expression of Elp5 have poor response to gemcitabine treatment. 2. In melanoma, Elp1 promotes the translation of HIF1A protein, leading to resistance of tumor cells to targeted therapeutic drugs such as BRAF V600E inhibitors.	Cancer: intestinal, breast, melanoma, medulloblastoma, gallbladder Function: remodels tumor microenvironment & immune evasion; drives multi-drug resistance (gemcitabine, BRAF V600E inhibitors); biomarkers for early low-Elp5/Elp1 status Prospect: sensitization / reversal of drug resistance.
FTSJ1	methyltransferase	tRNA U34	Providing potential molecular targets for the development of anti-tumor metastasis drugs: reducing the ability of cancer cells to respond to oxidative stress and inhibiting tumor metastasis.	Cancer: melanoma Function: anti-ROS / reduces oxidative stress response; metastasis suppressor markers Prospect: anti-metastasis drug development.

This table compares and summarizes the modification methods of different tRNA modifying enzymes mentioned above and their roles in clinical practice and cancer.

In summary, ALKBH8 affects the ROS detoxification network and protein translation by regulating tRNA modification, thereby influencing the occurrence and development of cancer. This suggests its potential application value in cancer treatment, which deserves further exploration.

### DNMT2 mediates tRNA codon-specific translation in cancer

DNMT2 (TRDMT1) is an RNA cytosine methyltransferase that converts cytosine (C) in tRNA to 5-methylcytosine (m^5^C), primarily targeting the C38 site. This modification can affect the complementary pairing between tRNAs and codons, thereby regulating the accuracy and efficiency of translation. When the modification function of DNMT2 is abnormal, it may lead to codon translation errors, thereby regulating the occurrence and development of cancer; thus, DNMT2 is a potential target for cancer treatment [73, 213]. DNMT2 is a highly conserved tRNA Asp methyltransferase that maintains cellular function. In cancer, mutations in the DNMT2 gene can affect its catalytic activity. For example, the E63K mutation doubles enzyme activity, whereas mutations such as G155S, L257V, R371H, and G155V significantly reduce or completely decrease enzyme activity. These mutations affect protein synthesis by altering tRNA binding ability or methylation, which may promote tumorigenesis. These findings provide important information for studying the mechanism of action of DNMT2 in tumorigenesis and offer potential targets for cancer treatment and diagnosis [214].

In cancer cells, DNMT2 affects codon-specific translation by modifying tRNA. For example, 5-azacytidine-mediated RNA immunoprecipitation (Aza IP) technology can be used to identify C > G translocations at cytosine residues targeted by DNMT2, thereby recognizing specific methylated cytosines in target RNA. In addition, 5-azacytidine can inhibit RNA methylation at the DNMT2 target site, thereby affecting the function of tRNA [215]. When DNMT2 is absent, FTO expression increases, leading to a decrease in the m6A methylation level of TNFSF10, which in turn upregulates TNFSF10 expression and significantly inhibits the proliferation and metastasis of DNMT2-deficient liver cancer cells. These findings suggest that DNMT2 may indirectly affect the biological behavior of liver cancer cells. Moreover, the absence of DNMT2 may also activate the intracellular apoptotic signaling pathway, allowing bortezomib to more effectively induce apoptosis in liver cancer cells [216]. In addition, DNMT2 affects the development of breast cancer, cervical cancer, osteosarcoma, glioblastoma and other cancers [217].

These findings suggest that DNMT2 affects protein synthesis and cellular function by regulating tRNA modifications and codon-specific translation, thereby influencing the development of cancer. Therefore, DNMT2 and its regulated tRNA modification pathway may become new targets for cancer therapy.

### METTL1 mediates tRNA m^7^G in cancer

METTL1 is a key tRNA methyltransferase located in the region of chromosome 12 (12q13-14), which is frequently amplified in various cancers and closely associated with tumorigenesis [90, 218]. METTL1 modifies the m^7^G site of tRNA to affect the translation efficiency of specific codons, thereby regulating the occurrence and development of cancer.

During the occurrence and development of tumors, the METTL1/WDR4 complex modifies the tRNA subpopulation that decodes m^7^G-dependent codons, promotes stable ribosome extension, and increases the cell cycle and translation efficiency of oncogenic mRNA, thereby promoting cancer progression [219, 220]. For example, in HCC, METTL1-mediated tRNA m^7^G modification significantly increases the translation of target mRNA with high-frequency m^7^G-related codons. METTL1 knockdown can reduce the level of m^7^G-modified tRNA in HCC cells and inhibit cell proliferation, migration, and invasion [221, 222].

From the perspective of drug resistance, the EGFR gene is a key target of METTL1, and its mRNA has a high m^7^G-related codon frequency. METTL1 enhances the function of tRNA corresponding to these codons, thereby promoting the translation efficiency of EGFR mRNA, increasing EGFR protein expression, and activating downstream signaling pathways such as the Akt and p44/42 MAPK pathways [222]. Activation of the EGFR pathway enhances the proliferation ability of liver cancer cells, inhibits apoptosis, and allows cells to survive and proliferate under treatment with lenvatinib, ultimately leading to the development of drug resistance [223, 224]. Moreover, METTL1/WDR4 selectively upregulates the translation of ECM remodeling-related genes through m^7^G tRNA modification, driving osteosarcoma resistance to doxorubicin. This mechanism suggests that inhibiting METTL1 may reverse drug resistance and provide a new strategy for combination chemotherapy [225]. In addition, METTL1 provides a new therapeutic intervention strategy against oral squamous cell carcinoma (OSCC) resistance by enhancing overall mRNA translation and stimulating oxidative phosphorylation (OXPHOS) [102, 226]. From a clinical feasibility perspective, drug resistance risk can be predicted, and treatment plans can be guided by detecting METTL1 expression or m^7^G modification levels; METTL1 inhibition can reverse drug resistance and be used as a targeted development inhibitor to enhance efficacy in combination with chemotherapy/targeted drugs. However, it is necessary to address issues such as specificity, standardization of detection, and response to multichannel drug resistance, which is expected to promote the precise treatment of tumors.

In various cancers, METTL1 affects tumor occurrence and progression by regulating tRNA codon-specific translation. For example, in esophageal squamous cell carcinoma (ESCC), METTL1 promotes tumor progression through the regulation of RPTOR gene translation. In ESCC cells METTL1 knockdown, the expression of RPTOR protein is reduced, leading to a decrease in mTORC1 activity, which specifically manifests as a decrease in the phosphorylation levels of the downstream target proteins p4EBP1 and pS6K1 of mTORC1 [227]. In cholangiocarcinoma (ICC), METTL1 affects the expression of cell cycle- and EGFR pathway-related genes by regulating the translation of oncogenic mRNA. Mechanistically, genes whose translation efficiency is reduced after METTL1 is knocked out have more m^7^G tRNA decoding codons [228, 229]. In prostate cancer, METTL1 promotes tumorigenesis through the biogenesis of tRNA-derived fragments, while its deletion leads to the loss of m^7^G modification, inhibiting tumor growth [230]. In breast cancer, METTL1-mediated m^7^G tRNA modification is necessary for codon recognition during mRNA translation [231]. For example, METTL1-mediated m^7^G tRNA modification can drive the progression and metastasis of thyroid carcinoma by regulating the codon-specific translation of TNF-α [232]. METTL1-mediated m^7^G modification affects codon translation and is also associated with various other cancers, such as lung cancer, leukemia, nasopharyngeal carcinoma, neuroblastoma, and gastric cancer [233–237].

### TRMT12 modifies the G37 site on tRNA in cancer

TRMT12 (also known as TYW2) is a tRNA modification enzyme that affects codon-specific translation by modulating the G37 site of tRNA, thereby regulating the occurrence and development of cancer. Research has shown that the transcriptional silencing of TRMT12 is associated with promoter CpG island hypermethylation in colorectal cancer (CRC). TRMT12 inactivation leads to the depletion of wybutosine derivatives (oHyW and o^2^yW), resulting in the loss of G37 site modification, which is crucial for maintaining the accuracy of ribosome reading frames. Therefore, the inactivation of TRMT12 triggers an increase in ribosomal -1 frameshift events, leading to the incorrect reading of codons and a decrease in translation efficiency [238].

Changes in the modification at the G37 site may affect the interaction between tRNAPhe and codons, increase the stability of codon‒codon interactions, and prevent code shift errors during translation. In terms of drug resistance, the downregulation of TRMT12 leads to an increase in imG-14 levels, which becomes the main modification of tRNAPhe in paclitaxel-resistant strains. Knocking out TRMT12 in HeLa cells leads to the accumulation of imG-14 and reduces the efficacy of paclitaxel. Therefore, low TRMT12 expression not only promotes cancer survival but also enhances resistance to paclitaxel treatment [239]. From a clinical feasibility perspective, detecting the mRNA or protein levels of TRMT12 in tumor tissues can be used to screen for patients at high risk for drug resistance and guide treatment plan adjustments (such as combining other drugs to reverse drug resistance). Targeted drugs (such as TRMT12 activators) can be developed to address the modification abnormalities caused by low TRMT12 expression, restore its normal expression to correct tRNA modification imbalance, and enhance the killing effect of paclitaxel on tumor cells. In addition, the combined inhibition of abnormal modifications such as imG-14 synthesis (such as by interfering with its upstream metabolic pathways) may further increase chemotherapy sensitivity.

In early colorectal cancer patients, high methylation of the TRMT12 promoter is significantly associated with poor overall survival and is an independent prognostic predictor. Cell model experiments have shown that colorectal cancer cells lacking TRMT12 exhibit enhanced migration ability and epithelial mesenchymal transition (EMT) characteristics, which are associated with the susceptibility of tumor cells to metastasis [238]. From a clinical feasibility perspective, TRMT12 detection relies on mature DNA methylation technology, which can be achieved through tumor tissue samples. TRMT12 detection can assist in early patient risk stratification and guide personalized treatment (such as enhanced follow-up or adjuvant chemotherapy); however, it is necessary to address the limitations of relying on tissue samples (such as exploring liquid biopsy) and validate methods in multicenter cohorts to improve specificity. In the future, TRMT12 is expected to be combined with other biomarkers to construct predictive models or develop targeted therapies, thereby promoting precise diagnosis and treatment. In addition, TRMT12 affects the occurrence and development of breast cancer and head and neck squamous cell carcinoma [173].

These results indicate that TRMT12 affects codon-specific translation by modulating the G37 site modification of tRNA, thereby regulating cancer progression and drug resistance. The epigenetic regulatory mechanism of its expression provides a new target for cancer treatment.

### TRMT61A mediates tRNA m^1^A in cancer

TRMT61A affects codon-specific translation by modulating the m^1^A modification of tRNA, thereby regulating the occurrence and development of cancer. In hepatocellular carcinoma (HCC), the methyltransferase complex formed by TRMT61A and TRMT6 is highly expressed, leading to a significant increase in the m^1^A methylation of tRNA, especially on tRNAAla (AGC) and tRNAGlu (CTC). This modification improves the translation efficiency of corresponding codons, increases the translation of peroxisome proliferator activated receptor δ (PPARδ), and is associated with a poor prognosis in HCC patients. Therefore, targeting the TRMT6/TRMT61A complex may be an effective strategy for treating HCC [9]. In addition, the depletion of TRMT6/TRMT61A in C6 glioma cells reduced cell proliferation and increased cell death, which could be partially rescued by the overexpression of tRNAi (Met) [240]. Moreover, the knockout or downregulation of TRMT61A resulted in a significant decrease in the m^1^A modification of tRNA. In the absence of the TRMT61A gene, the m^1^A modification of tRNA is reduced, thereby inhibiting ACL translation, blocking cholesterol biosynthesis, and weakening the tumor-killing function and proliferation ability of CD8⁺ T cells. CD8⁺ T cells play crucial roles in the immune defense against solid tumors such as melanoma and lung cancer, as well as hematological tumors such as leukemia. Abnormal expression of TRMT61A may lead tumor cells to escape immune surveillance and promote tumor progression [16]. High expression of TRMT61A can increase the m^1^A modification of tRNA, improve the recognition efficiency of corresponding codons in MYC mRNA, and promote MYC protein synthesis. MYC, as a transcription factor, activates the transcription of PD-L1, thereby upregulating its expression. As an immune checkpoint protein, highly expressed PD-L1 can bind to PD-1 on the surface of T cells, inhibit T-cell activity, help tumor cells evade immune surveillance, and promote tumor immune escape [241]. In head and neck squamous cell carcinoma (HNSCC), TRMT61A-mediated tRNA m^1^A58 modification directly regulates the translation of MYC mRNA through codon decoding, thereby affecting tumor occurrence and development [241].

TRMT6/TRMT61A plays a carcinogenic role in bladder cancer (BLCA) and participates in the regulation of the cell stress response. However, its specific mechanism in bladder cancer needs further study [242]. In addition, TRMT61A may play a role in various cancers, such as testicular germ cell tumors, but its specific mechanism is still unclear and deserves further exploration. These studies will help reveal the role of TRMT61A in cancer and provide new targets for cancer treatment [243–246].

### NSUN2 mediates tRNA codon-specific translation in cancer

NSUN2 is a tRNA modification enzyme that affects codon-specific translation by regulating the m^5^C modification of tRNA, thereby regulating the occurrence and development of cancer. Research has shown that the expression level of NSUN2 is elevated in various cancers and that its mediated tRNA m^5^C modification can significantly affect the efficiency and accuracy of protein translation.

The expression of NSUN2 gradually increases in normal thyroid, poorly differentiated thyroid cancer (PDTC), and undifferentiated thyroid cancer (ATC) tissues. Knocking out NSUN2 leads to a decrease in m^5^C methylation levels of cytoplasmic tRNAs, which have species and secondary structure preferences; this decrease manifests as a reduction in m^5^C modifications of certain specific tRNAs (such as tRNA Leu CAA and CAG). A decrease in the methylation rate of m^5^C modification sites can affect the secondary structural stability and function of tRNA, thereby affecting codon recognition and protein translation. For example, m^5^C modification of stable tRNA mediated by NSUN2 can more effectively recognize genes rich in TTG codons, thereby promoting the protein translation of these genes. This mechanism is crucial for the synthesis of oncogene-related proteins such as c-Myc, BCL2, RAB31, JUNB, and TRAF2, which play critical roles in tumor cell growth and proliferation. Therefore, NSUN2 regulates the m^5^C modification of tRNA, affects codon-specific translation, and thereby regulates cancer progression and drug resistance [247]. In addition, NSUN2 plays important roles in various cancers, such as esophageal cancer, liver cancer, gastric cancer, ovarian cancer, and head and neck squamous cell carcinoma, by affecting tRNA modification and may become a valuable target for cancer treatment and a diagnostic marker for cancer [145, 248–250].

### Modification of tRNA U34 position in cancer

The elongator complex plays an important role in cancer. From the perspective of the interaction between microorganisms and human immunity, Elp3 is the catalytic subunit of the Elongator complex, is responsible for modifying the U34 site of cytoplasmic tRNA, and is crucial for maintaining the mcms²U modification of tRNA. In *Aspergillus niger*, knocking out the elp3 gene leads to the loss of mcms²U in tRNA, which in turn affects hyphal growth, the production of biofilm-associated extracellular polysaccharides (GAGs), adhesion ability, and virulence. When human immune function is low, *Aspergillus fumigatus* infection may trigger an inflammatory response, and a persistent inflammatory microenvironment is associated with the occurrence and development of cancer. In addition, in *Aspergillus niger*, Elp3 deficiency upregulates the expression of genes related to amino acid metabolism and overexpression of the transcription factor CpcA. If this abnormal regulation of amino acid metabolism occurs in the tumor microenvironment, it may affect the proliferation and survival of tumor cells. Therefore, Elp3 may play an important role in the development of the tumor microenvironment by regulating amino acid metabolism through affecting tRNA modification [251].

In addition, dysfunction of the Elongator complex is closely related to the progression of various tumors. In intestinal epithelial cells, the Wnt signaling pathway can upregulate Elp3 expression, promote Sox9 protein expression, and maintain cancer stem cell subpopulations. In breast cancer, the oncogenic expression of T protein in multiple oncoviruses can increase the expression of Elp3 and Ctu1/2, promote the translation of DEK oncoproteins, and upregulate the characteristics of the invasion-promoting transcriptome. In melanoma, Elp3 modifies tRNA to affect the decoding of specific codons in HIF1A mRNA, thereby promoting the translation of HIF1A protein. The increase in HIF1A protein expression enables melanoma cells to acquire invasive features and drug resistance, leading to resistance of tumor cells to targeted therapeutic drugs such as BRAF V600E inhibitors. In breast cancer, Elp3 is associated with tRNA modification and the IRES-dependent translation of LEF1 to maintain metastasis in breast cancer. In pediatric medulloblastoma, germline mutations in Elp1 and sustained activation of the Sonic Hedgehog signaling pathway increase patient susceptibility to disease [203, 206, 252]. In gallbladder cancer, patients with low expression of Elp5 respond poorly to gemcitabine treatment. In medulloblastoma, the loss of mutations in Elp1 can cause instability of the Elongator complex, loss of tRNA modifications, and trigger codon-dependent translational reprogramming and unfolded protein responses. This, in conjunction with PTCH1 somatic changes and activation of the SHH signaling pathway, increases the susceptibility of individuals to tumors and affects their molecular characteristics [253–255].

In addition to the Elongator complex, FTSJ1-mediated U34 methylation (forming mcm^5^Um, i.e., Um34) can increase the efficiency of selenocysteine (Sec) insertion into protein sequences, thereby increasing the translation level of redox-regulated selenoproteins such as GPX4. However, this process may reduce the oxidative stress tolerance of cancer cells and inhibit tumor metastasis. Therefore, FTSJ1 and its mediated Um34 modification can serve as potential targets for antitumor metastasis drugs. In melanoma cells, exposure to selenium and reactive oxygen species (ROS) can increase FTSJ1-mediated Um34 formation, promote stress-responsive selenoprotein synthesis, alleviate ROS damage, and support cell survival under oxidative conditions [256].

### Role of codon-specific translation in cancer

Codon-specific translation exhibits both cancer type specificity and dynamic changes with tumor progression stage. In terms of cancer-specific differences, studies have shown that different types of cancer affect the same tissue in terms of different patterns of synonymous codon usage changes. For example, in breast cancer, invasive ductal carcinoma (IDC), invasive lobular carcinoma (ILC), and mixed invasive ductal and lobular carcinoma (IDLC) are associated with a significant increase in the use of the glycine codon GGT. However, in IDC and IDLC, but not in ILC, the change in GGT use preference is related to the change in GGC use preference. These findings indicate that different types of breast cancer have different codon usage preferences [257]. In various cancers, the expression of multiple codons of tRNAArg (such as CGT, AGA, CGG, and CGA) is generally upregulated, with tRNAArg (CGT) and tRNAArg (AGA) being highly expressed in renal clear cell carcinoma (KIRC) and associated with a poor prognosis. Other codons, such as tRNAThr (ACA) and tRNAPro (CCA), are downregulated in KIRC and are associated with a poor prognosis. TRNAVal is upregulated in 9 types of cancer and downregulated in 5 types of cancer, reflecting the cancer specificity of its function. Moreover, the upregulated tRNA in prostate cancer corresponds mainly to the AGC codon of serine (Ser) and the ACC codon of threonine (Thr), with little difference in codon usage compared with that in normal prostate tissue (low MSE value). The upregulated tRNA coverage in cholangiocarcinoma is wider, including not only Ser AGC and Thr ACC, which are involved in prostate cancer, but also the AAA codon of lysine (Lys) and the CTT codon of leucine (Leu). There is a significant difference in codon usage patterns between cholangiocarcinoma tissue and normal bile duct tissue (high MSE value). This reflects the heterogeneity of codon-specific translation among different cancer types [258].

There are also some examples in terms of tumor progression stages. For example, through survival analysis, it was found that the degree of change in codon usage (MSE value) between normal and tumor tissues of patients is correlated with prognosis; the greater the change is, the higher the mortality rate of patients. This finding indirectly suggests that as the tumor progresses (such as with increasing malignancy), abnormal changes in codon preference may intensify, but their specific stage-specific patterns still need further research for verification [257]. Moreover, TRMT6 is highly expressed in colorectal cancer (CRC), stabilizing the TRMT6-TRMT61A complex, enhancing the m^1^A modification of tRNA-Lys-TTT-1-1, and promoting histone translation with an AAA/AAG codon preference, thereby driving cell cycle and tumor progression. High expression of TRMT6 is associated with a poor prognosis in CRC patients, and its translational regulation is key for CRC cell proliferation, indirectly suggesting that a preference for AAA/AAG codons may continue to play a role in CRC progression. The specific correlation mechanism is worthy of further exploration [179]. These findings provide a basis for considering codon preference features in personalized cancer treatment and exploring their potential research value.

## Discussion and outlook

In this review, we explore the structure, modification enzymes, and mechanisms of tRNA, as well as their roles in tumor development. tRNA modifications, such as m^6^A, m^5^C, m^1^G, Ψ, D, I, and 2’-O, can influence tumor progression by modulating codon preference during translation. This is often mediated by tRNA modification enzymes, which can induce stop codon rewriting and ribosomal frameshifting or alter base status and translation efficiency. Common tumor-associated modifications include m^7^G and m^5^G.

From the perspective of tumor diagnosis, treatment, and prognosis, tRNA modification enzymes can serve as biomarkers and regulate drug resistance. For example, modulating the codon frequency to regulate EGFR translation can maintain resistance to lenvatinib in hepatocellular carcinoma. However, challenges remain in fully understanding the mechanisms underlying tRNA-mediated codon translation in tumor development. Many promising tRNA modification enzymes, such as PUS1, the Trm family, the TRM10 family, TRMT61A-C, and the NSUN family, require further investigation. Therefore, in terms of future clinical relevance and basic research, standardized detection reagents for tRNA modification and related molecules can be developed for early diagnosis, prognostic stratification, and drug resistance risk prediction in cancer; moreover, specific tRNA modification enzyme inhibitors can be developed and combined with existing chemotherapy/targeted drugs to reverse drug resistance, or drug combinations can be adjusted based on tumor cell tRNA modification profiles and codon preferences. Future basic research requires systematic mapping of the correlation between tRNA modification profiles and codon usage frequency in different cancers to clarify the specific axis of action for various tRNA modification enzymes and tRNA codon target genes; explore the effect of tumor microenvironment regulation on tRNA function; explore the cross-interactions between tRNA modifications and other forms of epigenetic regulation; explore the role of noncoding RNA; and develop tRNA modification editing tools and high-throughput screening platforms. By combining clinical translation with basic research, this field is expected to offer breakthroughs in the precision oncology by providing new strategies for overcoming drug resistance and improving prognosis.

In addition to affecting tumors, tRNA modification enzymes also play significant roles in neurological, reproductive, and hematological diseases. Thus, studying tRNA and its modifications is of great importance.

In conclusion, tRNA modifications are closely related to tumor development and progression. The mechanisms discussed here highlight the potential of tRNA-mediated codon-specific translation in influencing disease outcomes. This field holds great potential for further exploration, which will contribute to advancing our understanding of tumor biology and ultimately benefit human health.

## Conclusion

In conclusion, our review highlights the critical role of tRNA modifications and their enzymes in tumor development and progression. These modifications, including m^6^A, m^5^C, m^1^G, and others, can influence tumor behavior by modulating codon preference and translation efficiency. Despite emerging insights into the underlying mechanisms, such as ribosomal frameshifting and stop codon rewriting, many questions remain unanswered. The potential of tRNA modifications as biomarkers and therapeutic targets in cancer, as well as their broader implications in other diseases, underscores the importance of further research in this field. Understanding these mechanisms will not only advance our knowledge of tumor biology but also pave the way for novel diagnostic and therapeutic strategies, ultimately benefiting human health.
